# Soil enzyme profile analysis for indicating decomposer micro‐food web

**DOI:** 10.1002/imt2.161

**Published:** 2024-01-02

**Authors:** Wen Xing, Ning Hu, Zhongfang Li, Liangshan Feng, Weidong Zhang, Gerhard Du Preez, Huimin Zhang, Dongchu Li, Shunbao Lu, Scott X. Chang, Qingwen Zhang, Yilai Lou

**Affiliations:** ^1^ Guangxi Key Laboratory of Health Care Food Science and Technology, School of Food and Biological Engineering Hezhou University Hezhou China; ^2^ Institute of Environment and Sustainable Development in Agriculture Chinese Academy of Agricultural Sciences Beijing China; ^3^ Liaoning Academy of Agricultural Sciences Shenyang China; ^4^ Institute of Applied Ecology Chinese Academy of Sciences Shenyang China; ^5^ Unit for Environmental Sciences and Management North‐West University Potchefstroom South Africa; ^6^ Institute of Agricultural Resources and Regional Planning Chinese Academy of Agricultural Sciences Beijing China; ^7^ Jiangxi Normal University Nanchang China; ^8^ Department of Renewable Resources University of Alberta Edmonton Alberta Canada

**Keywords:** co‐occurrence network, decomposition channel, food web, microbiome, nematode, protozoa, soil exoenzyme

## Abstract

Highly diverse exoenzymes mediate the energy flow from substrates to the multitrophic microbiota within the soil decomposer micro‐food web. Here, we used a “soil enzyme profile analysis” approach to establish a series of enzyme profile indices; those indices were hypothesized to reflect micro‐food web features. We systematically evaluated the shifts in enzyme profile indices in relation to the micro‐food web features in the restoration of an abandoned cropland to a natural area. We found that enzymatic C:N stoichiometry and decomposability index were significantly associated with substrate availability. Furthermore, the higher Shannon diversity index in the exoenzyme profile, especially for the C‐degrading hydrolase, corresponded to a greater microbiota community diversity. The increased complexity and stability of the exoenzyme network reflected similar changes with the micro‐food web networks. In addition, the gross activity of the enzyme profile as a parameter for soil multifunctionality, effectively predicted the substrate content, microbiota community size, diversity, and network complexity. Ultimately, the proposed enzymic channel index was closely associated with the traditional decomposition channel indices derived from microorganisms and nematodes. Our results showed that soil enzyme profile analysis reflected very well the decomposer food web features. Our study has important implications for projecting future climate change or anthropogenic disturbance impacts on soil decomposer micro‐food web features by using soil enzyme profile analysis.

## Highlights

- Soil enzyme profile analysis for indicating decomposer micro‐food web features was first proposed.
- Diversity indication utilities for contrasting enzyme profiles were different.
- Protozoa channel index and enzymatic channel index were proposed.

## Graphical abstract

This study proposed the approach of a “soil enzyme profile analysis” whereby a series of enzyme profile indices were hypothesized to comprehensively reflect the micro‐food web features. Using a case study on the restored natural area of a post‐arable system, we systematically evaluated the shifts in enzyme profile indices in relation to the micro‐food web features. +, −, ↑, and ↓ represent positive correlation, negative correlation, increase, and decrease trends, respectively.

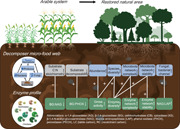

## INTRODUCTION

**Figure 1:**
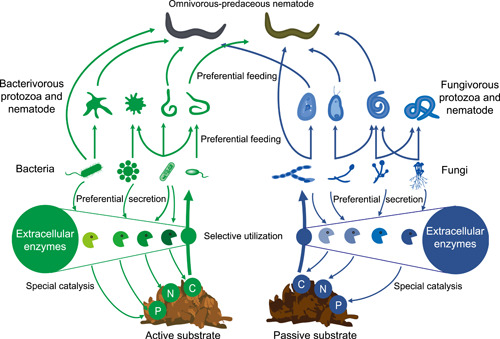
Classic structure of soil decomposer micro‐food web and its conceptual links to exoenzyme profile. The green and blue colors represent the bacteria‐ and fungal‐based energy flow channels, respectively.

The soil decomposer food web plays a critical role in mediating biogeochemical cycling and sustaining ecosystem functions [1]. In the soil micro‐food web, bacteria and fungi predominantly contribute to the decomposition of soil substrates [2]. Microfauna, represented mainly by protozoa and nematodes, prey on microbes and thus regulate microbial populations [3, 4]. Also, nematode communities are species‐rich, multitrophic, and occupy a central position in the soil micro‐food web [5, 6]. Ecological indices calculated using nematode community data are extensively used to characterize soil micro‐food web [7]. Therefore, there is a growing interest in studying substrate‐based micro‐food web consisting of microbes and microfauna (Figure 1) [8]. Traditionally, the soil micro‐food web is characterized by substrate quantity and quality (stoichiometry and decomposability), the abundance, diversity, and species composition of microbiota, and dominant energy channels (i.e., fungal‐ and bacterial‐based channels). Additional interest has been focused on network complexity and stability, which represent major determinants of ecosystem functioning underpinned by the micro‐food web [9, 10].

Exoenzymes are extracellular enzymes that play a major role in soil food webs [11]. At least 500 exoenzymes, which catalyze the rate‐limiting steps of decomposition and mediate energy flow in soil food webs, are thought to primarily originate from soil microorganisms [12, 13]. Catalytic reactions of extracellular exoenzymes are substrate‐specific, while soil substrates are highly variable spatially and temporary [14] (Figure 1). Therefore, the spatial and temporal variabilities of soil substrates strongly influence exoenzyme activities. However, the enzymatic stoichiometry theory states that microorganisms can adapt to substrate changes by modifying the type of exoenzymes produced [15]. Consequently, ratios of exoenzyme activities can reflect substrate composition [14]. Additionally, the extracellular exoenzyme production of microorganisms is identity‐specific (Figure 1). For instance, the production of peroxidase must be carried out by specialist saprotrophic fungi, for example, *Basidiomycota* [11, 16]. Therefore, shifts in soil microbial community composition may result in changes in exoenzyme production [16]. Moreover, the feeding of some microfauna on microbes is preferential (Figure 1). For example, the protozoa *Acanthamoeba castellanii* preferentially feeds on the fungi *Coprinus cinerea* [17] and the bacterivorous nematode *Caenorhabditis elegans* preferentially feeds on the bacterium *Escherichia coli* [18]. Hence, the exoenzyme production pattern may be modified by the species composition of microfauna.

Ecological indices that provide comprehensive information on decomposer micro‐food web is lacking [5, 8]. Some of the key characteristics of the micro‐food web as discussed above may be studied by a specific soil exoenzymatic profile analysis. Here, we first propose a methodology for soil exoenzyme profile analysis, which is expected to be an integrated measure of decomposer micro‐food web features and to predict soil micro‐food web status (Table 1). In this study, we assessed the application of soil exoenzyme profile analysis by using a case study based on the conversion of an arable system to a natural area. Multiple studies have indicated distinct differences in substrate status, community size, species diversity, dominant decomposition channel type, and network properties of soil food web between arable and restored natural systems [19, 20, 21]. We hypothesized that a series of indices derived from soil exoenzyme profile analysis would be strongly associated with the changes in decomposer micro‐food web features (Table 1) with the conversion of cropland to a natural ecosystem.

**Table 1:** The proposed soil exoenzyme profile indices for indicating the decomposer micro‐food web features.

Exoenzyme profile index	Calculation	Implications for decomposer micro‐food web features
Stoichiometry index	Ratios of activities of exoenzymes acquiring elements	Linked to substrate stoichiometry and element constraint
Carbon decomposability index	The ratio of carbon hydrolase activity to oxidase activity	Indicating carbon bioavailability of substrate
Channel index	The ratio of fungal‐ to bacterial‐derived exoenzyme activities	Reflecting the dominance of fungal‐ versus bacterial‐based decomposition channel
Exoenzyme composition	The first principal coordination score (PCo1) calculated by the Bray–Curtis dissimilarity matrix	Associated with the taxa composition of microbiota in micro‐food web
Gross activity	The average of standardized exoenzyme activity	Greater gross exoenzyme activity indicates greater substrate quantity, community size, diversity, and network complexity
Diversity	Alpha diversity, incorporating species richness and evenness (Shannon Index and Pielou Evenness Index) for a specifically selected exoenzyme profile	Higher diversity of exoenzyme profile indicates a more diverse microbiota community
Network complexity	Complexity parameters (such as average degree, average clustering coefficient, etc.) derived from co‐occurring network analysis	The exoenzyme profile with a greater network complexity corresponds to the more complex microbiota network
Network stability	Stability parameters (such as robustness and vulnerability) derived from co‐occurring network analysis	The exoenzyme profile with a higher network stability corresponds to the more stable microbiota network

## RESULTS

### Decomposer micro‐food web features

**Figure 2:**
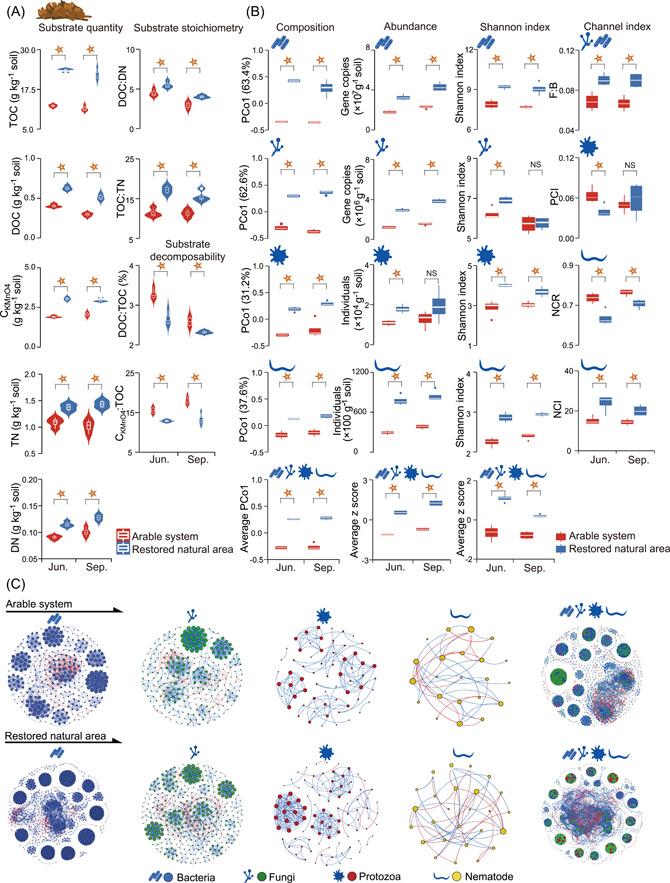
Decomposer micro‐food web features in the arable system and restored natural area. (A) Soil substrate quantity, substrate stoichiometry, and decomposability, (B) characteristics of soil microbiota community, and (C) co‐occurrence networks of soil microbiota community. Significance analysis was performed using the *T* test. NS, *p* > 0.05; 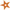, *p* < 0.05. $C_{{\mathrm{KMnO}}_{4}}$, KMnO_4_‐oxidized C; DN, dissolved nitrogen; DOC, dissolved organic carbon; F:B, fungi:bacteria; NCI, nematode channel index; NCR, nematode channel ratio; PCI, protozoa channel index; PCo1, the first principal coordination score; TN, total nitrogen; TOC, total organic carbon. The average of PCo1 scores of the four functional groups (average PCo1), the average standardized abundance, or Shannon Index of the four functional groups (average *z* score).

Compared with the arable system, the restored natural area presented higher soil total organic carbon (TOC), dissolved organic carbon (DOC), KMnO_4_‐oxidized C $\left(C_{{\mathrm{KMnO}}_{4}}\right)$, total nitrogen (TN), and dissolved nitrogen (DN) contents of 92.9%, 63.4%, 50.9%, 33.2%, and 26.7%, respectively (Figure 2A). Furthermore, TOC:TN and DOC:DN increased, while DOC:TOC and $C_{{\mathrm{KMnO}}_{4}}$:TOC decreased, respectively. Significant differences across the two seasons were recorded for all these parameters and metrics.

The composition of each functional group and the whole microbiota community was significantly different under the restored natural area (Figure 2B). Specifically, the natural area had a 25.9% lower relative abundance of bacterial dominant taxa *Chloroflexi*, 31.8% higher fungal dominant taxa *Sordariomycetes*, 8.4% lower protozoa dominant taxa *Cerozoa*, and 9.1% lower nematode dominant taxa *Mesorhabditis* across two seasons, compared with the arable system (Figure S1).

The abundance of each functional group was significantly increased under the restored natural area in both seasons except for protozoa in September (Figure 2B). Across the two seasons, the abundances of bacterial, fungal, protozoan, and nematode communities were increased by 83.7%, 145.2%, 58.8%, and 140.1%, respectively. Also, the standardized abundance of the whole microbiota was significantly increased in the restored natural area in both seasons (Figure 2B).

Compared with the arable system, the restored natural area had 37.1%, 50.8%, 83.4%, and 50.1% higher richness of bacterial, fungal, protozoa, and nematode communities, respectively, across both seasons (Figure S2A). The evenness index of each functional group and the overall microbiota was higher in the natural area, and the difference was significant for bacteria, fungi, and nematode in June, and protozoa in September (Figure S2B). The Shannon Index of each functional group was significantly increased after the conversion of the arable system to a restored natural area in both seasons except for fungal diversity in September (Figure 2B). An average of 11.7%, 6.2%, 25.1%, and 30.2% increases across both seasons for bacterial, fungal, protozoa, and nematode communities, respectively, were found. Also, the standardized richness and Shannon Index of the whole microbiota community were significantly increased in both seasons (Figures S2B and 2B).

Compared with the arable system, the natural area had 33.2% and 48.9% higher fungi:bacteria (F:B) ratio and nematode channel index (NCI), respectively, and 10.5% less nematode channel ratio (NCR) across the two seasons (Figure 2B). The protozoa channel index (PCI) was 37.3% lower in June, but was not different in September in the natural area, in comparison with the arable system.

For the microbiota co‐occurrence networks (Figure 2C), comparing the natural area to the arable system, specific network complexity indices such as the average degree (avgK) and average clustering coefficient (avgCC) values significantly increased for the bacterial network (Table S1). For the fungal network, the average path distance (GD) value significantly increased, while the values of the other four indices did not change. Values of avgK and GD significantly increased for both the nematode and the whole micro‐food web networks. Robustness values of community networks of bacteria and nematode, as well as the whole micro‐food web, significantly increased, while vulnerability values decreased for bacteria and whole micro‐food web networks after the arable conversion to restored natural area (Table S1). Comparing the restored natural area to the arable system, more positive, negative links, and modules were detected for the microbiota co‐occurrence networks (Table S2).

### Exoenzyme profile indices

**Figure 3:**
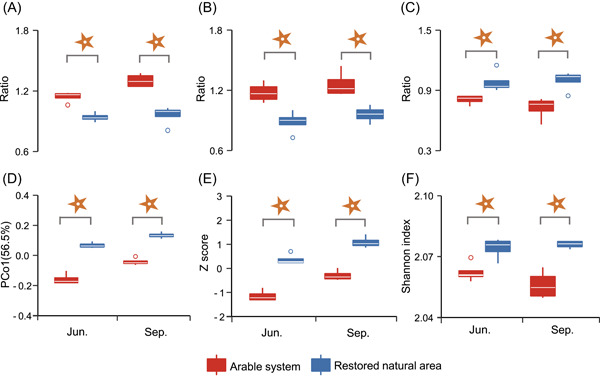
Soil exoenzyme profile indices based on exoenzymes involved in carbon and nitrogen cycling under arable system and restored natural area in two seasons. Significance analysis was performed by the *T* test. 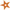, *p* < 0.05. (A) BG:NAG, (B) BG:PHOX, (C) NAG:LAP, (D) composition, (E) gross activity, and (F) diversity. BG, β‐1,4‐glucosidase; LAP, leucine aminopeptidase; NAG, β‐1,4‐*N*‐acetyl‐glucosaminidase; PCo1, the first principal coordination score; PHOX, phenol oxidase.

Compared with the arable system, β‐1,4‐glucosidase:β‐1,4‐*N*‐acetyl‐glucosaminidase (BG:NAG) and β‐1,4‐glucosidase:phenol oxidase (BG:PHOX) were significantly decreased, while β‐1,4‐*N*‐acetyl‐glucosaminidase:leucine aminopeptidase (NAG:LAP) was significantly increased under the restored natural area, on average two seasons (Figure 3A–C). For the exoenzyme profile composition indices, the first principal coordination (PCo1) scores showed significant differences between treatments in both seasons (Figure 3D). Specifically, the relative activities of α‐1,4‐glucosidase (AG), cellobiohydrolase (CB), and PHOX were increased by 2.2%, 3.4%, and 1.6% in the restored natural area, across both seasons, respectively (Figure S3). The gross exoenzyme activity and Shannon Index of the exoenzyme profile were also significantly elevated in the restored natural area (Figure 3E,F).

For the exoenzyme networks, greater values of exoenzyme network complexity parameters including avgK, avgCC, and connectedness (Con) were observed in the restored natural area than those in the arable system (Table S3). The robustness value of the exoenzyme network was significantly higher in the restored natural area.

### Correlations between exoenzyme profile indices and decomposer micro‐food web features

**Figure 4:**
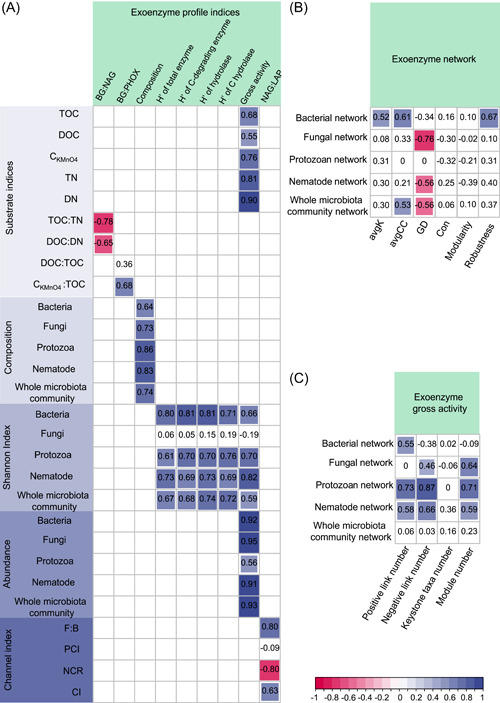
Correlations between soil exoenzyme profile indices and decomposer micro‐food web features. (A) Correlations between soil exoenzyme profile indices and the characteristics of soil substrate and microbiota community, (B) between network complexity and stability indices of exoenzyme profile and microbiota community, (C) between exoenzyme gross activity and network indices of microbiota community (*n* = 16) after the arable system converted to a natural system. Pearson correlation coefficients at *p* < 0.05 are indicated in red (negative) or in blue (positive). The size of the square is proportional to the absolute value of the correlation coefficient. The blank squares indicate that no correlation analysis was conducted. avgCC, average clustering coefficient; avgK, average degree; BG, β‐1,4‐glucosidase; $C_{{\mathrm{KMnO}}_{4}}$, KMnO_4_‐oxidized C; Con, connectedness; DN, dissolved nitrogen; DOC, dissolved organic carbon; F:B, fungi:bacteria; GD, average path distance; H′, Shannon Index; LAP, leucine aminopeptidase; NAG, β‐1,4‐*N*‐acetyl‐glucosaminidase; NCI, nematode channel index; NCR, nematode channel ratio; PCI, protozoa channel index; PHOX, phenol oxidase; TN, total nitrogen; TOC, total organic carbon.

BG:NAG presented a significant negative correlation with the substrate C:N stoichiometry indices (i.e., TOC:TN and DOC:DN) (Figure 4A). BG:PHOX was significantly and positively correlated with the decomposability index of $C_{{\mathrm{KMnO}}_{4}}$:TOC.

In terms of the PCo1 scores, the exoenzyme profile composition was significantly correlated with the composition of bacterial, fungal, protozoan, nematode groups, and the whole microbiota community (Figure 4A). The Shannon Index of exoenzyme profiles (comprising all eight of the selected exoenzymes) was significantly and positively correlated with the Shannon Index of each functional group (except fungi) and the whole microbiota communities. Similarly, there were significantly positive correlations between Shannon indices of C‐degrading exoenzyme, hydrolase, and C‐degrading hydrolase profiles with those of bacteria, protozoa, and nematode groups, and the whole microbiota communities. The Shannon Index of C‐degrading hydrolase showed the strongest correlations with microbiota communities among all types of reference exoenzyme profiles.

The avgK value of the exoenzyme network was positively correlated with the avgK value of the bacterial network (Figure 4B). The avgCC value of the exoenzyme network was positively correlated with the avgCC values of bacterial and whole microbiota networks. In addition, the robustness of the exoenzyme network was positively associated with the robustness of the bacterial network.

Gross activity of the exoenzyme profile was positively correlated with substrate quantity indices (i.e., TOC, DOC, $C_{{\mathrm{KMnO}}_{4}}$, TN, and DN contents), the abundance of each functional group and the whole microbiota community, and the Shannon Index of each functional group (except fungi) and the whole microbiota community (Figure 4A). The gross activity of the exoenzyme profile was also positively correlated with the number of positive links, negative links, and modules of bacterial, fungal, and protozoan and nematode networks (Figure 4C). NAG:LAP was positively correlated with F:B and NCI, and negatively with NCR, while exhibiting no association with PCI (Figure 4A).

## DISCUSSION

### Exoenzymatic stoichiometry and decomposability indices were linked to substrate quality of the micro‐food web

The stoichiometry and decomposability of substrates are key drivers of micro‐food web function [11]. In our case study, after the arable system was converted to a natural system, TOC:TN and DOC:DN were both increased, indicating the increased C availability but decreased substrate N availability for soil micro‐food web. Furthermore, $C_{{\mathrm{KMnO}}_{4}}$:TOC and DOC:TOC decreased, suggesting decreased substrate C decomposability. We found that BG:NAG was decreased under the restored natural area, and presented negative correlations with TOC:TN and DOC:DN. This indicated the usefulness of BG:NAG in evaluating the substrate C:N stoichiometry. Our results strongly supported the enzymatic stoichiometry theory [15] that soil microorganisms can adapt to nutrient deficiency by shifting the type of exoenzymes produced in favor of nutrient cycling and therefore boosting nutrient turnover. While only C:N stoichiometry was investigated in this study, numerous studies have also shown the close association between enzymatic C:P or N:P with substrate C:P or N:P [14, 22, 23]. Also, we observed that BG:PHOX was significantly decreased after the arable system was converted to the natural area and it exhibited significant positive correlations with $C_{{\mathrm{KMnO}}_{4}}$:TOC and DOC:TOC. This suggests that the enzymatic C decomposability index can indicate substrate C decomposability and is consistent with the result of previous studies reporting that microorganisms can modulate the relative allocation of resources into exoenzymes that target labile versus recalcitrant C in response to the change of substrate C decomposability [24].

Note that these exoenzymes were chosen for calculating the corresponding indices of the soil micro‐food web, because their catalyzed substrate components are thought to be the most abundant in respective relevant substrate complexes in most study cases [11, 15]. However, the utility of these indices may depend on the specific substrate background. For example, a previous study found that in the study with cellulose as the dominant C source in the soil food web, BG:NAG can effectively reflect the C versus N requirements of microorganisms, while the study focused on chitin, peptidoglycan, and protein as the main C source, BG:NAG cannot be used to indicate the microbial C versus N requirements [25]. As a result, appropriately selecting specific exoenzymes according to the specific substrates present is critical for improving the utility of these enzymatic indices for indicating changes in the substrate features of soil food web.

### Composition and diversity of exoenzymatic profile associated with the micro‐food web

Consistent with the results of multiple previous studies [19, 20, 21], we found that each functional group and the whole microbiota community exhibited greater diversity in terms of richness, evenness, and the Shannon Index in the restored natural area compared with the arable system. This is possibly due to increased substrate amount (e.g., increased TOC and TN as presented in this study), substrate complexity, and/or improved habitats (e.g., improved soil pH and aggregation) in the restored natural area benefit coexistence of multiple microbes [26, 27]. However, the exoenzyme richness did not differ between the arable system and the restored natural area for all the studied exoenzymes detected in both treatments. This possibly resulted from a study limitation with only eight exoenzymes included, all of which are common. Selecting a larger number of exoenzymes, also including rare ones, can be important for improving the indication utility of the exoenzyme profile. As we expected, the exoenzyme profile composition was significantly altered and the Shannon Index was higher in the restored natural area. There were also significant correlations between the composition and Shannon Index of the exoenzyme profile and the composition and Shannon Index of microbiota functional groups and the whole microbiota community. This showed the diversity‐indication utility of exoenzyme profile, which can firstly be attributed to different species of microorganisms performing distinct metabolic functions and producing distinct exoenzyme profiles [28]. Additionally, the specific trophic interaction of microbe–microfauna (protozoa and nematodes) accounted for the diversity correlation between exoenzymes and microfauna. In the present case study, there were significant positive correlations between microbe and microfauna diversities (Figure S4). On the one hand, microbial community structure can modify microfauna structure via bottom‐up effects [29]. Therefore, exoenzyme profile structure can be indirectly linked to microfauna community structure. On the other hand, microfauna community structure can regulate microbial community structure via top‐down effects, and in turn indirectly control the microbe‐derived exoenzyme profile structure [30].

As discussed previously, in our case study, the changed substrate stoichiometry and decomposability resulted in significant shifts in the structure of specific exoenzymes under the restored natural area. This can lead to confusing in the utility of exoenzyme profile evenness for indicating the microbiota diversity. To minimize this effect, we further selected specific subsets of the exoenzyme profile to assess the diversity indications of microbiota. We found a strong positive correlation between the Shannon Index of the C‐degrading hydrolase with the Shannon Index of most microbiota communities and the whole micro‐food web. This was possibly due to that compared with the C‐degrading hydrolase profile, the studied total enzyme, C‐degrading enzyme, hydrolase profiles were comprised of more types of exoenzymes, and thus the C‐degrading hydrolase profile was the least influenced by the substrate stoichiometry and decomposability among the studied enzyme profile [28]. These results indicate that when using exoenzyme evenness to evaluate microbiota diversity, selecting the appropriate exoenzyme sub‐profile is important with regard to the study area and research question(s). Alternatively, selecting a larger number of exoenzymes may be useful to increase the effectiveness of this approach.

### Network complexity and stability of exoenzymatic profile corresponding to those of the micro‐food web

Soil food web complexity is traditionally described based on trophic interactions among microbiota functional groups. The network with species as nodes and species–species relationships as links, is generally used to characterize complex ecological interactions such as predation, competition, and mutualism within the soil food web [20, 31, 32]. Using this network analysis, a series of parameters quantifying ecological network complexity and stability have been developed.

Because exoenzymatic secretion patterns can be distinct among different microbiota taxa, interactions between taxa may normally induce certain correlations of corresponding exoenzymes. For instance, assuming exoenzyme A1 (random naming) specifically originates from microbiota taxa B1 and exoenzyme A2 from taxa B2, then the interaction between B1 and B2 (assumed as a positive link) would cause a statistically positive correlation between A1 and A2. Higher biodiversity of soil food webs often supports a more complex ecological network [10], and a more complicated network usually shows greater stability. This is probably because the complexity indices such as connectivity and modularity are positively associated with the robustness and stability of complex systems [10]. In the present case study, we also found that compared with the arable system, as microbiota diversity increased under the restored natural area, the network complexity and stability of the soil micro‐food web also increased, as indicated by its greater avgK, avgCC, and GD, strengthened robustness, and weakened vulnerability. Additionally, although only eight types of exoenzymes were included in the co‐occurrence network analysis, higher avgK, avgCC, and Con values, and greater robustness were observed. This indicates that more complex and stable exoenzyme networks were present in the restored natural system compared with the arable system. Furthermore, we found avgK and avgCC of the exoenzyme network were positively correlated with avgK and avgCC of the biota network, respectively. Seemingly, there was a corresponding relationship between exoenzyme profile and micro‐food web structure in terms of their network properties.

### Exoenzymatic gross activity in relation to substrate quantity, community size, diversity, and network complexity of the micro‐food web

Soil microorganisms are known to acquire energy, carbon, and multiple nutrients for their growth through secreting exoenzymes to catalyze soil substrate (Figure 1). Typically, enzyme activity represents a key indicator of soil fertility and microbial function. In our case study, the increased soil organic carbon and nitrogen contents stimulated microbial growth, which in turn elevated the gross activity of exoenzymes after the conversion of the arable system to a restored natural area. Additionally, the stimulated microbial growth further promoted the growth of their predators (i.e., protozoa and nematodes) via the bottom‐up effects of trophic cascading [29]. This resulted in a significant increase in the abundance of protozoa and nematodes, and caused positive correlations between microbiota and their predators' abundances (Figure S4). As a result, the gross activity of the exoenzyme profile was positively correlated not only with soil carbon and nitrogen contents, but also with microbial, protozoa, and nematode abundances. This indicates that the gross activity of the exoenzyme profile can be used as an indicator to reflect substrate quantity and community size of the micro‐food web.

Because the gross activity of exoenzyme profile represents multiple metabolic functions (i.e., soil multifunctionality) of soil microorganisms [33], and that the capacity of soil biotic communities maintaining multifunctionality is strongly dependent on soil biodiversity [34, 35, 36, 37], we expected that there would be a strong association between exoenzyme gross activity and soil biodiversity. Indeed, we observed positive correlations between the gross activity of exoenzyme profile and the Shannon Index of multiple soil organisms (i.e., bacteria, protozoa, nematode, and whole microbiota community). This can be explained by the classic assumptions of “complementarity effect” and “selection effect” [38]. The complementarity effect is defined as the ability of different species to each utilize a different subset of the available niches, with the consequence that a multispecies assemblage will exploit a greater part of the resource than any singular species [39]. Therefore, a combination of different species may not only coexist, but also outperform any of them surviving in isolation [39]. Alternatively, the selection effect suggests that dominant species with particular traits can overperform specific functions in a community [40]. Apart from richness, evenness is also believed to favor the multifunctionality of a community. In an uneven community dominated by a few taxa, any organism is likely to interact with its kin [39]. Conversely, in a community with high species evenness, an organism will most likely interact with other functionally dissimilar organisms, which is helpful to promote synergistic interactions and thus boost soil multifunctionality [36].

The association complexity among biotic taxa provides a new dimension for understanding the positive effects of biodiversity on multifunctionality [9]. The gross activity of the exoenzyme profile thus can also reflect the network complexity of the micro‐food web, as indicated by our case study. This can be explained by the following three aspects. Firstly, the positive (e.g., facilitation) and negative (e.g., competition) interactions among different taxa drive ecosystem functions [9]. As presented in this case study, a more complex network may possess more numbers of positive and/or negative links (Table S1), suggesting more taxonomic interactions, which may exert positive influences on soil multifunctionality in terms of enzymatic gross activity. In addition, keystone taxa are highly connected taxonomic groups conferring greater biotic connectivity to a community [41]. In our study, more numbers of keystone taxa were identified in the restored natural area networks. These keystone taxa are important for maintaining network structure and thus multiple functions, which could positively affect soil multifunctionality [42]. Finally, a module in the network is a group of taxa which are highly connected among themselves, but less connected with taxa outside the group [31]. In the food web, functionally dissimilar groups often form different network modules, so that a community with more modular architectures may have more functional group diversity and stronger ability to provide multiple ecosystem functions [43]. Indeed, more numbers of modules were detected for the microbiota co‐occurrence networks after the conversion of the arable system to a restored natural area in our case study.

### Exoenzymatic channel index for reflecting decomposition channel structure of the micro‐food web

The energy flow in a soil food web consists mainly of bacterial‐ and fungal‐based channels [2]. The relative importance of these two channels represents a crucial parameter for predicting the functioning and sustainability of the food web [1]. As we expected, the decomposition channel of the restored natural area shifted towards fungal dominance relative to the arable system. This was indicated by enhanced F:B and NCI, and reduced NCR, which is consistent with previous reports [20, 44]. Traditionally, protozoa are thought to prey mainly on bacteria [17, 45]. However, recent studies pointed out that some protozoa (e.g., *A. castellanii*) prefer to feed on fungi [17]. On the basis of this information, here we first tried to propose the PCI for indicating the decomposition channel structure among different ecosystems. However, in the present study, the PCI value did not increase under the restored natural area when compared with the arable system. This indicated that the above‐mentioned view of some protozoa preferentially feeding on fungi may be questionable, and so the PCI calculation needs to be further improved in future studies. Alternatively, this can be explained by the limited sampling time points in this study. With respect to the PCI as an instant indicator, its response to the restored natural area might be seasonally varied and the response might be ineffective in indicating the structural change of gross decomposition at some specific sampling time points. Hence, further testing should be done on the PCI utility including additional sampling time points.

Considering that NAG is known to originate mainly from fungi [11], here we first proposed the enzymatic channel index calculated by the ratio of NAG to LAP. These two enzymes are involved in nitrogen cycling [15]. In the present study, NAG:LAP was observed to significantly increase after the conversion of arable land to a restored natural area, and to show strong positive correlations with F:B and NCI, and a negative correlation with NCR. This indicated the usefulness of NAG:LAP in reflecting the decomposition channel structure. Higher NAG:LAP means greater relative importance of fungal‐based decomposition pathways in the soil micro‐food web. Among the microbiota, microorganisms are known to dominantly contribute to the energy flow of the soil food web, and thus the channel indices derived from microorganisms such as F:B normally represent the basic criterion for indicating decomposition channel structure. Due to the longer reproductive period of higher trophic‐level organisms (e.g., nematodes), there may be a lag of several days to weeks before changes in faunal channel indices in response to microbial changes [5]. In contrast, soil exoenzymes produced by microorganisms can rapidly shift in response to environmental change. Consequently, the enzymatic channel index may be more effective in predicting the instant change in decomposition channel structure than other faunal channel indices.

## CONCLUSION

In this case study on the restored natural area of an arable system, we found close associations between the proposed soil enzyme profile indices and the corresponding parameters of the decomposer micro‐food web. Our data indicated that “soil enzyme profile analysis” can be a useful tool to comprehensively reflect decomposer micro‐food web features. However, its general utility needs to be further examined. Moreover, it is recommended that a sufficient number and appropriate type collection of enzymes should be considered based on the outcomes of the study.

To optimize and develop enzyme profile indices and to promote the application of the “soil enzyme profile analysis” for indicating changes in soil micro‐food web, the following two study directions need to be further strengthened in future work: (і) specific sources of soil exoenzymes: although many soil exoenzymes have been found and they are known to originate mainly from microbial secretion, our knowledge of the unique sources of exoenzymes remains limited. It will be helpful for us to better understand theoretical links between exoenzymes and the micro‐food web. For example, if some exoenzyme(s) are exclusively derived from soil microfauna, the relative activity of such exoenzyme(s) would be expected to indicate a vertical trophic structure of the micro‐food web; (ii) high‐efficient measurement of exoenzyme activities: the fluorescence detection technology has greatly enhanced measurement efficiency of soil exoenzyme activities, but the types of enzymes that can be measured by this technology remains limited. As mentioned above, the more enzymatic identities included in the reference “soil enzyme profile,” the better the predictive ability of diversity and network parameters related to the enzyme profile as part of the micro‐food web.

## METHODS

### Study site

A long‐term field experiment with an “arable system” and “a restored natural system” was selected for this study. The experiment was established in 1990 at the National Soil Quality Observation Experimental Station at Qiyang (26°45′ N, 119°52′ E) in Hunan Province, Southern China. This region belongs to a subtropical monsoon climate with a mean annual precipitation and temperature of 1250 mm and 18°C, respectively. The soil is classified as a Ferralic Cambisol (WRB Classification). The field experiment includes a series of treatments on soil use and nutrient management strategies with each plot area of 200 m^2^. Before initiating the field experiment, topsoil (0–20 cm) chemical properties were as follows: TOC = 6.7 g kg^–1^, TN = 1.07 g kg^–1^, available N (AN) = 79 mg kg^–1^, available phosphorus (AP) = 10.8 mg kg^–1^, and pH = 5.70.

### Experiment design

For this study, two treatments were selected, namely, a conventional cropping (i.e., arable) system and a restored natural area. There were four plots (25 m^2^ each) as replicates for each treatment. The arable system followed a rainfed wheat–maize rotation with conventional fertilization. Urea, superphosphate, and potassium chloride were applied at rates of 188 kg N ha^−1^ year^−1^, 41 kg P ha^−1^ year^−1^, and 78 kg K ha^−1^ year^−1^, respectively, for maize, and at rates of 165 kg N ha^−1^ year^−1^, 36 kg P ha^−1^ year^−1^, and 68 kg K ha^−1^ year^−1^, respectively, for wheat. Crop straw was removed after harvest. The vegetation was dominated by herbaceous plants, shrubs, and trees in the restored natural area.

### Soil sampling

Soil samples were collected in June and September 2021. At each sampling, six topsoil (0–15 cm) samples were collected from each subplot to form one composite sample. This composite sample was divided into three parts and sieved using the required mesh sizes. One part was stored at −80°C for soil genomic DNA extraction, while another part was air‐dried for soil property measurements. Finally, the remaining part was stored at 4°C for less than 1 week for soil moisture and exoenzyme activity measurements, as well as protozoa and nematode extractions.

### Substrate measurements

Soil TOC and TN contents were measured using an elemental analyzer (Vario EL V, Elementar). Since the soil was acidic and its carbonate content was neglected, we used total C content as an approximation of TOC. The KMnO_4_‐oxidized C was determined following the methods described by the previous study [46]. Soil DOC and DN were extracted using a 0.5 M K_2_SO_4_ solution. The supernatant was filtered through a 0.45 µm filter and the C and N concentrations in the filtrate were determined using an automated TOC/TN analyzer (multi‐N/C 3000, Analytik Jena).

The ratios of TOC to TN (TOC:TN) and DOC to DN (DOC:DN) were calculated as the substrate C:N stoichiometry. The SOC decomposability was assessed considering the proportion (%) of DOC to TOC and KMnO_4_‐oxidized C to TOC.

### Bacterial and fungal community analysis

Abundances of bacteria and fungi were assessed using their gene copy numbers. Soil genome DNA was extracted from homogenized 0.5 g subsamples using the FastDNA SPIN Kit for Soil (MP Biomedicals) following the manufacturer's instructions. Bacterial and fungal ribosomal RNA (rRNA) gene copy numbers were quantified by quantitative polymerase chain reaction (PCR) using a StepOnePlus Real‐Time PCR System (Applied Biosystems). Primer set 27F/519R [47] and ITS1/ITS4 [48] were used to target 16S and ITS rRNA genes, respectively. Standard curves were generated using 10‐fold serial dilutions of linearized recombinant plasmids for each quantitative PCR (qPCR) run. The qPCR assay was performed in triplicate in a 20‐μL reaction volume containing 10 μL of 2× concentration SYBR Premix Ex Taq (premix of dNTP Mixture, SYBR Green I, Mg^2+^, Tli RNaseH, Takara Ex Taq HS), 0.4 μL of forward and reverse primers, 0.4 μL of ROX Reference Dye (50×), 2 μL of template DNA, and 6.8 μL of autoclaved distilled water. The ratio of fungal gene to bacterial gene copy numbers (F:B) was calculated as a decomposition channel index.

High‐throughput sequencing was performed for bacterial and fungal community composition analysis. We amplified the bacterial 16S rRNA gene with the targeting primer set 338F/806R [49] and the fungal ITS1 rRNA gene with the targeting primer set ITS1F/ITS2R [48]. Purified amplicons were sequenced on an Illumina MiSeq platform (2 × 300, pair end). Sequencing data were processed using the open‐source Quantitative Insights into Microbial Ecology 2 (QIIME2, version 2021.4) pipeline [50]. Sequences were quality filtered, denoised, and the chimeras removed using the DADA2 plugin [51]. Next, the sequences were clustered into amplicon sequence variants (ASVs). Taxonomic identities were assigned to ASVs by the classify‐sklearn naïve Bayes taxonomy classifier in the feature‐classifier plugin [52] using the SILVA version 132 database for bacteria [53] and the UNITE version 8.3 database for fungi [54]. Alpha‐diversity metrics (i.e., richness, Pielou Evenness Index, and Shannon Index) were calculated based on the rarefied data at a minimum number of sequences [55, 56].

### Protozoan community analysis

The abundances of major protozoa groups including flagellates, amoebae, and ciliates were determined using the most‐probable number method [57]. Briefly, 2 g fresh soil was suspended in 18 mL sterile water and shaken at 180 rpm for 30 min. A threefold dilution series was added into 96‐well microtiter plates in quadruplicates. The plates were incubated at 25°C in the dark. After 4, 7, and 11 days, the wells were examined for the presence of protozoa using a Nikon Eclipse TS100 inverted optical microscope. The abundances of the major protozoa groups were determined and expressed as the number of individuals g^–1^ dry soil.

High‐throughput sequencing was used to characterize the protozoan community. We amplified the 18S rRNA gene with the targeting primer set TAReuk454FWD1/TAReukREV3 [58]. The sequencing data process for the protozoa community was similar to that of the bacteria and fungi. ASVs were taxonomically classified by blasting against the Protist Ribosomal Reference database (version 4.14) [59]. The protozoa community was semi‐quantitatively assayed based on identified protozoan sequences [60, 61], which were selected from the obtained ASV table. We assigned protozoa ASVs to functional groups of bacterivores and fungivores according to their feeding habits [60, 62]. The same alpha‐diversity metrics used previously were applied to estimated protozoan diversity based on the rarefied data at a minimum number of sequences. This study is the first to propose the PCI, calculated as the ratio of the relative abundance of fungivorous to bacterivorous protozoa, as a new decomposition channel index.

### Nematode community analysis

Nematodes were extracted from 100 g fresh soil (field condition) using the shallow dish method [63]. Next, nematodes were killed and fixed by adding a 4% formaldehyde solution heated to 65°C. In each sample all extracted nematodes were counted and identified to genus level using macro slides and an optical microscope (Olympus BX50). The nematode community was classified into the following four trophic groups: bacterivores, fungivores, omnivores–predators, and herbivores. The first three trophic groups (i.e., free living nematodes) included in the decomposer food web were used for the nematode community analysis, from which a minimum of 100 nematodes were chosen to calculate diversity indices (i.e., richness, Pielou Evenness Index, and Shannon Index). The rarefied data were further used to calculate the NCR as follows: NCR = B/(B + F), where B and F are the abundance of bacterivores and fungivores, respectively [6]. Additionally, nematodes were assigned into functional guilds that are based on life history characteristics [5]. More information on the functional guild classification of nematodes can be found at http://nemaplex.ucdavis.edu/. The NCI, which is a measure of the primary decomposition pathway (bacterial vs. fungal), was calculated as follows: NCI = 100 × [0.8 × Fu_2_/(3.2 × Ba_1_ + 0.8 × Fu_2_)], where Ba_1_ and Fu_2_ are the abundances of bacterivorous colonizer–persister (cp) group 1 and fungivorous cp group 2, respectively [5].

### Overall microbiota characterization

On the basis of the relative abundance of taxa, we calculated Bray–Curtis dissimilarity matrices for bacteria, fungi, and protozoa using ASV data, and for nematodes using genus level data with the vegdist function in R (version 4.1.1). Next, a principal coordinate analysis (PCoA) was conducted, and the PCo1 score was used as the indicator of community composition. The average of PCo1 scores of the four functional groups was used as an index of the whole microbiota community composition. Then, the *z* score approach [64] and the average standardized abundance, richness, Pielou Evenness Index, and Shannon Index of the four groups were used to reflect on the whole microbiota community abundance and diversity, respectively.

### Microbiota co‐occurrence network analysis

To visualize potential interactions among all individual members of bacteria, fungi, protozoa, nematode, and of the whole microbiota community, we constructed co‐occurrence networks for the corresponding communities. For the whole microbiota community, the relative abundance table was established by combining bacterial, fungal, and protozoa ASVs and nematode genera. Pairwise Spearman correlation matrixes were calculated, with cutoffs for the absolute value of correlation coefficient >0.6. *p* Values had a 0.05 cutoff. Networks for the arable system and the restored nature area were constructed independently. To evaluate the network complexity, we calculated five topological parameters: avgK, avgCC, GD, Con, and modularity in the subgraph function via the igraph package in R (version 4.1.1). Furthermore, robustness and vulnerability were used to evaluate network stability. Robustness is defined as the proportion of the remaining members in this network after random or targeted nodes removal [65]. Vulnerability is defined as the relative contribution of each node to the global efficiency which measures how fast information spreads within the network [41]. We measured the robustness of each network when 50% of random nodes were removed, and calculated the maximal vulnerability of nodes in the network [10]. Moreover, we calculated the number of positive or negative links for different networks. Finally, the topological roles of individual nodes in the network were determined by the threshold values of *Zi* and *Pi*. Nodes were classified into four categories: module hubs (*Zi* > 2.5), network hubs (*Zi* > 2.5 and *Pi* > 0.62), connectors, and peripherals (*Zi* < 2.5 and *Pi* < 0.62). The connectors, module hubs, and network hubs were categorized as keystone nodes [31]. In theory, greater avgK indicates greater number of positive or negative links between species in community; greater avgCC suggests that species were more extensively divided into several different components; greater GD means greater overall independence among species; higher Con suggests greater proportion of positive (or negative) links from all possible links in the network; greater modularity implies greater separation strength of species clusters within the network; strengthening robustness, and weakening vulnerability indicates a stabilizing network [10, 66].

### Exoenzyme activity measurements

Four hydrolytic C‐acquiring exoenzymes (AG, BG, CB, and xylosidase [XS]), two hydrolytic N‐acquiring exoenzymes (NAG and LAP), and two oxidative exoenzymes (PHOX and peroxidases [PEOX]) were selected to form a reference exoenzyme profile. Hydrolytic exoenzyme activities were assayed using standard fluorometric techniques [67]. The soil suspension was supplemented with 7‐amino‐4‐methylcoumarin (MUC for LAP) or 4‐methylumbelliferone (MUB for other hydrolytic exoenzymes) to establish standard curves. Each sample was assayed with six replicates in 96‐well microplates and incubated in the dark at 25°C for 3 h. Then the fluorescence was measured using a fluorescence spectrometer (Beckman Coulter DTX 880) with 365 nm excitation and 450 nm emission filters. Oxidase activities were measured spectrophotometrically using the substrate l‐3,4‐dihydroxy‐phenylalanine. For PEOX, 0.3% H_2_O_2_ was added. The microplates were incubated in the dark at 25°C for 20 h and then the absorbance was quantified at 450 nm. The units for all exoenzyme activities were nmol g^−1^ soil h^−1^.

### Exoenzyme profile analysis

We calculated a series of exoenzyme profile indices as follows: the ratio of BG to NAG (BG:NAG) was calculated as an enzymatic stoichiometry index to study substrate C:N stoichiometry; the ratio of BG to PHOX (BG:PHOX) was calculated as the enzymatic C decomposability index; the ratio of NAG to LAP was calculated (NAG:LAP) as the enzymatic channel index to reflect the dominance of fungal‐ versus bacterial‐based decomposition channels in the soil decomposer micro‐food web. This is possible since NAG is thought to be mainly secreted by fungi, while bacteria are a major source of soil LAP activity [11]. Furthermore, the mean of all standardized exoenzyme activities was used as the gross activity index of the whole exoenzyme profile. Since large differences in the order of magnitude of specific activities existed among the eight exoenzymes, we first conducted a simple mathematical transformation by dividing the original data of each exoenzyme by its maximum value observed in the data set of samples, and then multiplying it by 100 [21]. Thereafter, based on the proportions of the eight exoenzymes, the PCoA was conducted and the PCo1 scores were used as the indicator of exoenzyme composition. Since all eight exoenzymes were detected in every sample, we did not calculate richness, but instead calculated Shannon diversity of the whole exoenzyme profile (eight exoenzymes), C‐degrading exoenzymes (four C hydrolases and two oxidases), hydrolase (four C hydrolases and two N hydrolases), and C hydrolase (four exoenzymes), using the formula *H*′ = (−∑*Pi*[ln *Pi*]), where *Pi* was the proportion of a particular exoenzyme activity to the sum of activities of all exoenzymes [21]. As described above, a correlation network approach was used to visualize potential interactions among all individual members of the specific exoenzyme profile, and then the network complexity and stability parameters were calculated in both treatments.

### Statistical analysis

All statistical analyses were performed in R version 4.1.1 (R Development Core Team 2021). A *T* test was used to evaluate effects of the restoration of cropland to the natural area on soil decomposer micro‐food web characteristics and exoenzyme profile indices in comparison to the arable system. Relationships between exoenzyme profile indices and corresponding micro‐food web characteristics, between soil microbes (bacteria and fungi) with substrate and microfauna (protozoa and nematode) after the conversion of an arable system to a natural area were evaluated by a Pearson correlation analysis. Statistical significance was set at *α* = 0.05.

## AUTHOR CONTRIBUTIONS

Yilai Lou conceived the ideas and designed the research. Weng Xing, Ning Hu, Gerhard Du Preez, Scott X. Chang, and Yilai Lou drafted the manuscript. Zhongfang Li, Liangshan Feng, Weidong Zhang, Huimin Zhang, Dongchu Li, and Shunbao Lu performed the experiments. All the authors read and approved the final manuscript.

## CONFLICT OF INTEREST STATEMENT

The authors declare no conflict of interest.

## Supporting information


**Figure S1**. Relative abundances of top 10 taxa of (A) bacteria, (B) fungi, (C) protozoa, and (D) nematode community under treatments of arable systems and restored natural areas in two seasons.
**Figure S2**. Richness (A) and evenness (B) of soil microbiota community (i.e., bacteria, fungi, protozoa, nematode, and the whole microbiota community) in arable system versus restored natural area.
**Figure S3**. The relative standardized activities of eight exoenzymes in arable system versus restored natural area in two seasons.
**Figure S4**. Correlations between the characteristics of bacterial and fungal communities with the characteristics of substrates and microfauna community after the arable system converted to a natural system.

**Table S1**. Network complexity and stability indices (means ± SD) of soil microbiota community in arable system and restored natural area.
**Table S2**. The number of positive and negative links, keystone taxa and modules of bacteria, fungal, protozoa, nematode, and the whole microbiota community networks in an arable system versus a restored natural area.
**Table S3**. Network complexity and stability indices (means ± SD) based on eight selected exoenzymes involved in carbon and nitrogen cycling under arable system and restored natural area in two seasons.

## Data Availability

The raw sequence data reported in this paper have been deposited in the Genome Sequence Archive in BIG Data Center, Beijing Institute of Genomics (BIG), Chinese Academy of Sciences, under accession numbers CRA009970, CRA009972, and CRA009974 that are publicly accessible at https://ngdc.cncb.ac.cn/gsa. The data and scripts used are saved in GitHub https://github.com/spinagexingwen/Xing2023iMeta. Supplementary materials (figures, tables, scripts, graphical abstract, slides, videos, Chinese translated version, and update materials) may be found in the online DOI or iMeta Science http://www.imeta.science/.
