# Supplementary material for: Soil enzyme profile analysis for indicating decomposer micro‐food web
Source: Imeta. 2024 Jan 2;3(1):e161. doi: 10.1002/imt2.161 (PMC10989158; doi:10.1002/imt2.161)
Supplement: Supplementary file 1 — Figure S1. Relative abundances of top 10 taxa of (A) bacteria, (B) fungi, (C) protozoa, and (D) nematode community under treatments of arable systems and restored natural areas in two seasons. Figure S2. Richness (A) and evenness (B) of soil microbiota community (i.e., bacteria, fungi, protozoa, nematode, and the whole microbiota community) in arable system versus restored natural area. Figure S3. The relative standardized activities of eight exoenzymes in arable system versus restored natural area in two seasons. Figure S4. Correlations between the characteristics of bacterial and fungal communities with the characteristics of substrates and microfauna community after the arable system converted to a natural system. [file IMT2-3-e161-s002.docx]

**Supporting information to**

# **Soil enzyme profile analysis for indicating decomposer micro-food web**

**Running title:** Soil enzyme profile analysis for indicating decomposer micro-food web

Wen Xing^1, 2#^, Ning Hu^1#^, Zhongfang Li^1^, Liangshan Feng^3^, Weidong Zhang^4^, Gerhard Du Preez^5^, Huimin Zhang^6^, Dongchu Li^6^, Shunbao Lu^7^, Scott X. Chang^8^, Qingwen Zhang^2*^, Yilai Lou^2*^

^1^Guangxi Key Laboratory of Health Care Food Science and Technology, School of Food and Biological Engineering, Hezhou University, Hezhou 542899, China

^2^Institute of Environment and Sustainable Development in Agriculture, Chinese Academy of Agricultural Sciences, Beijing 100081, China

^3^Liaoning Academy of Agricultural Sciences, Shenyang 110161, China

^4^Institute of Applied Ecology, Chinese Academy of Sciences, Shenyang 110164, China

^5^Unit for Environmental Sciences and Management, North-West University, South Africa

^6^Institute of Agricultural Resources and Regional Planning, Chinese Academy of Agricultural Sciences, Beijing 100081, China

^7^Jiangxi Normal University, Nanchang 330022, China

^8^Department of Renewable Resources, University of Alberta, Alberta 510632, Canada

***Corresponding address:**

Prof. Qingwen Zhang and Prof. Yilai Lou

Institute of Environment and Sustainable Development in Agriculture, Chinese Academy of Agricultural Sciences, Beijing 100081, China

E-mail: [zhangqingwen@caas.cn](mailto:zhangqingwen@caas.cn) and [louyilai@caas.cn](mailto:louyilai@caas.cn)

**^#^These authors contributed equally to this work.**

**This file contains:**

Figure S1 Relative abundances of top 10 taxa of (A) bacteria, (B) fungi, (C) protozoa, and (D) nematode community under treatments of arable system and restored natural area in two seasons.

Figure S2. Richness (A) and evenness (B) of soil microbiota community (i.e., bacteria, fungi, protozoa, nematode, and the whole microbiota community) in arable system vs. restored natural area.

Figure S3. The relative standardized activities of eight exoenzymes in arable system vs. restored natural area in two seasons.

Figure S4. Correlations between the characteristics of bacterial and fungal communities with the characteristics of substrates and microfauna community after the arable system converted to a natural system.

**Figure S1** Relative abundances of top 10 taxa of (A) bacteria, (B) fungi, (C) protozoa, and (D) nematode community under treatments of arable system and restored natural area in two seasons. Values are means of four replicate subplots.

**Figure S2** Richness (A) and evenness (B) of soil microbiota community (i.e., bacteria, fungi, protozoa, nematode, and the whole microbiota community) in arable system vs. restored natural area. Significance analysis was performed by a T test in both seasons. NS, *p* > 0.05; **
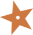
**, *p* < 0.05.

**Figure S3** The relative standardized activities of eight exoenzymes in arable system vs. restored natural area in two seasons. Values are means of four replicate subplots. Abbreviations: α-1,4-glucosidase (AG), β-1,4-glucosidase (BG), cellobiohydrolase (CB), xylosidase (XS), β-1,4-N-acetyl-glucosaminidase (NAG), leucine aminopeptidase (LAP), phenol oxidase (PHOX), peroxidases (PEOX).


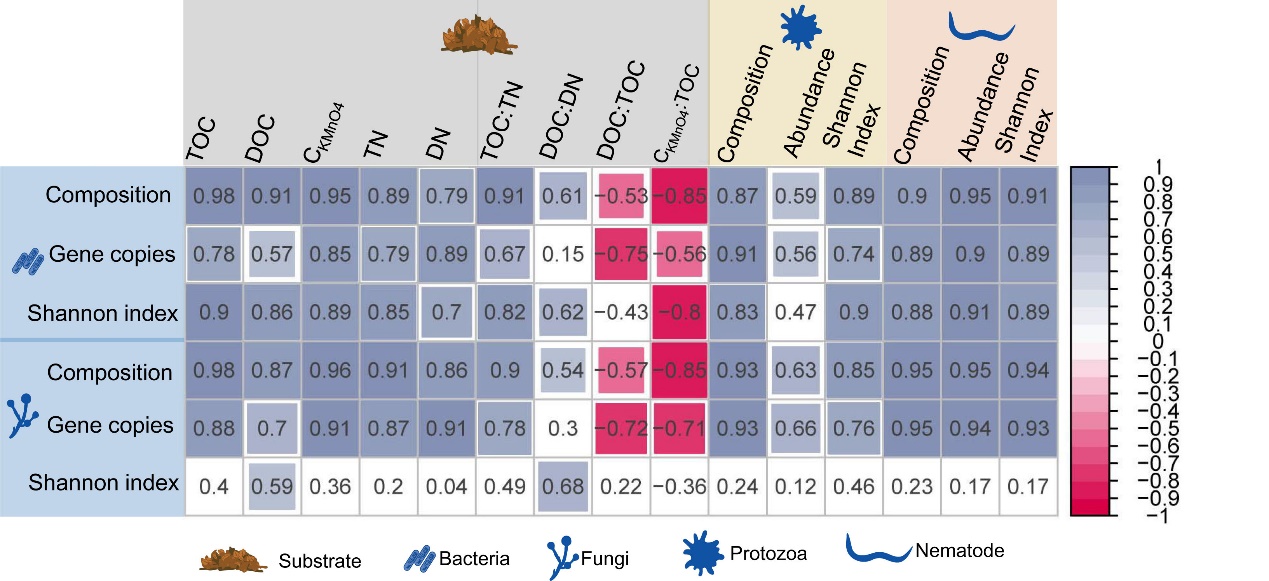
**Figure S4** Correlations between the characteristics of bacterial and fungal communities with the characteristics of substrates and microfauna community after the arable system converted to a natural system (*n* = 16). Pearson correlation coefficients at *p* < 0.05 are indicated in red (negative) or in blue (positive). The size of the square is proportional to the absolute value of the correlation coefficient. Abbreviations: total organic carbon (TOC), dissolved organic carbon (DOC), KMnO_4_-oxidized C (C_KMnO4_), total nitrogen (TN), dissolved nitrogen (DN).
